# Immune Checkpoint Inhibitors after Radiation Therapy Improve Overall Survival Rates in Patients with Stage IV Lung Cancer

**DOI:** 10.3390/cancers15174260

**Published:** 2023-08-25

**Authors:** Hidekazu Tanaka, Kazushi Ueda, Masako Karita, Taiki Ono, Yuki Manabe, Miki Kajima, Koya Fujimoto, Yuki Yuasa, Takehiro Shiinoki

**Affiliations:** Department of Radiation Oncology, Yamaguchi University Graduate School of Medicine, 1-1-1 Minamikogushi, Ube 755-8505, Yamaguchi, Japan

**Keywords:** radiation therapy, immune checkpoint inhibitor, lung cancer, stage IV

## Abstract

**Simple Summary:**

This study aimed to evaluate whether there is a difference in the overall survival (OS) rates of patients with stage IV lung cancer who underwent radiation therapy (RT) depending on the presence or absence of immune checkpoint inhibitors (ICIs). Eighty patients with stage IV lung cancer were enrolled. Patients treated with ICIs had significantly better OS rates than those not treated with ICIs (*p* < 0.001). The 6-month OS rates in patients treated with and without ICIs were 76.3% and 34.5%, respectively. The group that received ICI therapy after RT had a significantly better OS rate than the group that received ICI therapy prior to RT (6-month OS: 94.7% vs. 40.0%, *p* < 0.001). In the multivariate analysis, ICI use after RT was a significant factor for OS (*p* < 0.001). Our results suggest that ICI administration after RT may prolong the OS of patients with stage IV lung cancer.

**Abstract:**

This exploratory and retrospective study aimed to evaluate whether there is a difference in the overall survival (OS) rates of patients with stage IV lung cancer who underwent radiation therapy (RT) depending on the presence or absence of immune checkpoint inhibitors (ICIs) and the timing of their use. Eighty patients with histologically confirmed stage IV lung cancer were enrolled, and ICIs were administered to thirty (37.5%). ICIs were administered before RT and after RT in 11 and 20 patients, respectively. The median follow-up period was 6 (range: 1–37) months. Patients treated with ICIs had significantly better OS rates than those not treated with ICIs (*p* < 0.001). The 6-month OS rates in patients treated with and without ICIs were 76.3% and 34.5%, respectively. The group that received ICI therapy after RT had a significantly better OS rate than the group that received ICI therapy prior to RT (6-month OS: 94.7% vs. 40.0%, *p* < 0.001). In the multivariate analysis, performance status (0–1 vs. 2–4) and ICI use after RT were significant factors for OS (*p* = 0.032 and *p* < 0.001, respectively). Our results suggest that ICI administration after RT may prolong the OS of patients with stage IV lung cancer.

## 1. Introduction

The advent of immune checkpoint inhibitors (ICIs) has revolutionized the treatment of locally advanced or metastatic lung cancer [1,2,3]. The PACIFIC trial reported excellent results with durvalumab after definitive chemoradiotherapy for stage III non-small-cell lung cancer (NSCLC) [4,5,6]. Several reports, such as the KEYNOTE-189, 407, and Impower150 studies, show that the concomitant use of ICIs in metastatic NSCLC improves overall survival (OS) rates and progression-free survival rates [7,8,9]. For extensive-stage small-cell lung cancer (SCLC), the usefulness of atezolizumab in the IMpower133 study [10,11] and durvalumab in the CASPIAN study [12,13] was demonstrated.

Although the abscopal effect phenomenon in patients treated with radiation therapy (RT) has long been recognized, it is extremely rare. However, Postow et al. reported an abscopal effect in patients with malignant melanoma treated with ipilimumab and RT [14], evoking renewed attention to the abscopal effect phenomenon. At the same time, there is great anticipation for RT to activate the immune system and enhance efficacy when combined with ICIs. Nevertheless, the ideal timing for combining RT and ICIs remains unclear.

Therefore, we evaluated whether there was a difference in the overall survival (OS) rates of patients with stage IV lung cancer who underwent RT, considering the use of ICI therapy and the timing of its administration.

## 2. Materials and Methods

This retrospective study was approved by the Institutional Review Board of Yamaguchi University Hospital and performed in accordance with the principles of the Declaration of Helsinki. Written informed consent was obtained from all patients before RT.

Patients who underwent RT at our hospital between June 2015 and December 2021 were included. The eligibility criteria were patients who received RT during the target period, had histologically proven stage IV lung cancer, and were followed up for at least one month after RT. Patients undergoing treatment for other cancers were excluded. Notably, patients with driver mutation-positive NSCLC often respond in the long term to molecularly targeted agents. Thus, patients in which epidermal growth factor receptor or anaplastic lymphoma kinase tyrosine kinase inhibitors were used during the course of treatment were excluded, as the presence or absence of these could significantly impact the results.

RT was administered to lesions that were symptomatic or likely to become symptomatic. The patient data were extracted at the time of RT. For cases where RT was performed on multiple lesions, the data at the time of the first RT were extracted. RT was performed using TrueBeam or TrueBeam STx (Varian Medical Systems, USA). The biologically effective dose (BED) was calculated to evaluate the treatment with various dose fractionations. The survival period was calculated from the start date of RT. The OS rate was calculated using the Kaplan–Meier method, and the log-rank test was used to compare the two groups. Univariate and multivariate analyses using the Cox proportional hazards model were performed to explore prognostic factors for OS. The parameters used for the univariate analysis were age (<70 vs. ≥70 years), pathology (NSCLC vs. SCLC), BED (≤39 vs. >39 Gy), dose per fraction (≤3 vs. >3 Gy), other metastases (present vs. absent), Eastern Cooperative Oncology Group performance status (0–1 vs. 2–4), and ICI use after RT (present vs. absent). Parameters with *p* values less than 0.05 were entered into the multivariate analysis. Statistical significance was set at *p* < 0.05. Patients who received ICI therapy before and after RT were excluded from the assessment when comparing the groups that received ICI therapy before RT with those who received it after RT.

## 3. Results

### 3.1. Patients

Eighty patients met the inclusion criteria. Sixty-nine (86.3%) were male and eleven (23.8%) were female. The histological cancer types were adenocarcinoma, squamous cell carcinoma, pleomorphic cell carcinoma, large-cell carcinoma, NSCLC, not otherwise specified, SCLC, and large-cell neuroendocrine cancer in 31 (38.8%), 19 (23.8%), 1 (1.3%), 1 (1.3%), 4 (5.0%), 21 (26.3%), and 3 (3.8%) patients, respectively. The irradiated sites were the bone, brain, mediastinum, cervical lymph nodes, pleura, and adrenal glands in 44 (55.0%), 21 (26.3%), 7 (8.8%), 4 (5.0%), 3 (3.8%), and 1 (1.3%) of the patients, respectively. The median BED was 39 (range: 27.3–72.0 Gy). Approximately 80% of the patients had other metastatic lesions outside of the RT field. The patient characteristics are shown in Table 1.

ICIs were administered to 30 (37.5%) patients. ICIs were administered before and after RT in 11 and 20 patients, respectively. One of these patients received ICI therapy before and after RT. The ICIs used in the group treated with ICI therapy before RT were pembrolizumab in seven cases, atezolizumab in three cases, and nivolumab plus ipilimumab in one case. The ICIs used in the group receiving ICI therapy after RT were pembrolizumab in thirteen cases, nivolumab in three cases, nivolumab plus ipilimumab in two cases, atezolizumab in one case, and durvalumab in one case. Table 2 presents the patient characteristics of the groups that received and those that did not receive ICIs.

### 3.2. Overall Survival Rates

Sixty-two patients died during the follow-up period. Among them, 60 patients died of lung cancer, and the remaining 2 died of other causes. Regarding the patients who died of other causes, one died of senility and the other of pneumonia. Eighteen patients were censored when they were transferred to other facilities for end-of-life care. The median follow-up period was 6 months (range: 1–37 months). The 6-month OS rate for all patients was 50.5%, and the median survival time (MST) was 7 months.

**Figure 1:**
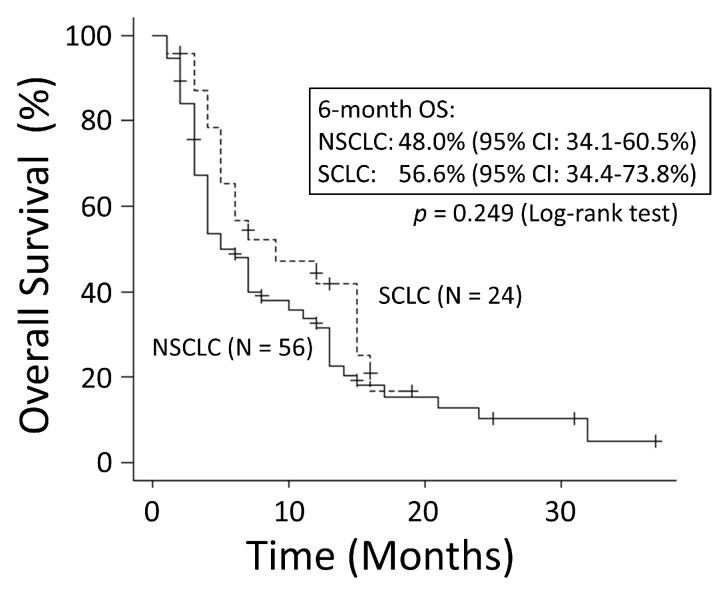
There was no significant difference in overall survival (OS) between non-small-cell lung cancer and small-cell lung cancer patients. CI: confidence interval.

**Figure 2:**
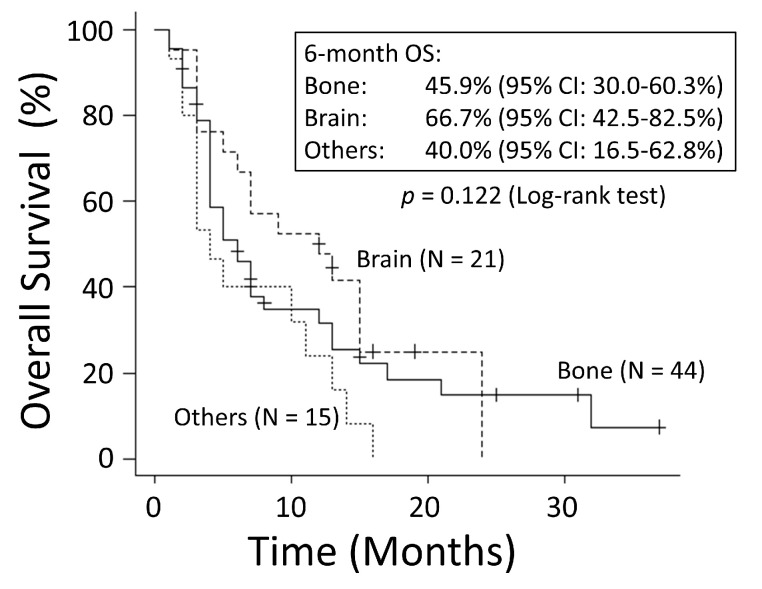
The good performance status (PS) group (PS ≤ 1) had significantly better overall survival (OS) than the poor PS group (PS ≥ 2). CI: confidence interval.

**Figure 3:**
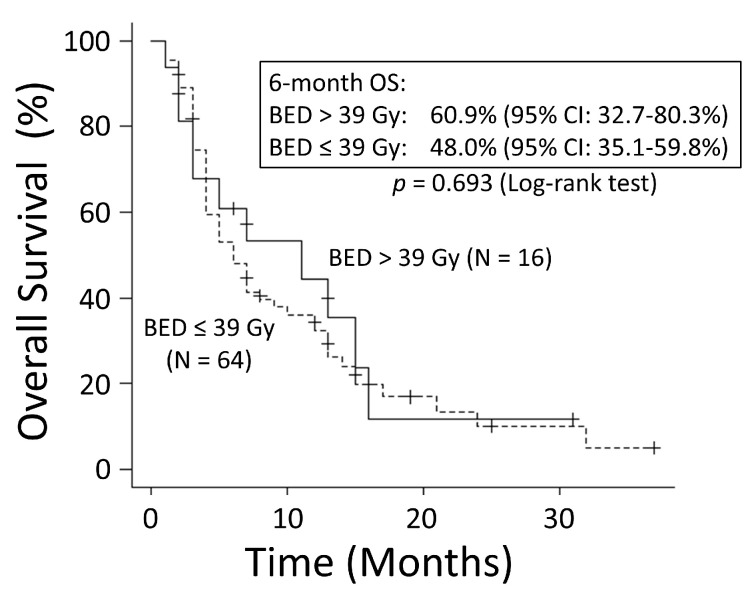
When the patients were divided into two groups based on their biologically effective dose (BED) values, either greater than the median BED of 39 Gy or ≤39 Gy, there was no significant difference in overall survival (OS) between the two groups. CI: confidence interval.

**Figure 4:**
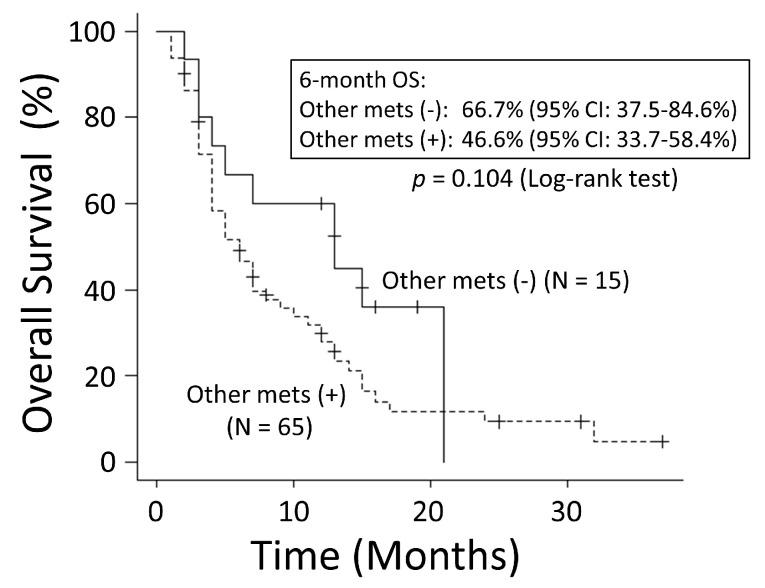
There was no significant difference in overall survival (OS) according to different irradiation sites. CI: confidence interval.

**Figure 5:**
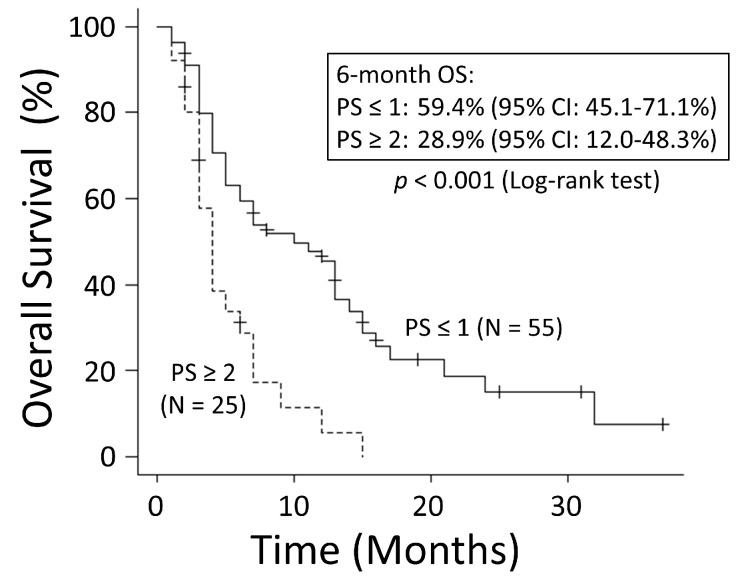
There was no significant difference in overall survival (OS) between the groups with and without metastases other than at the irradiated sites. CI: confidence interval.

**Figure 6:**
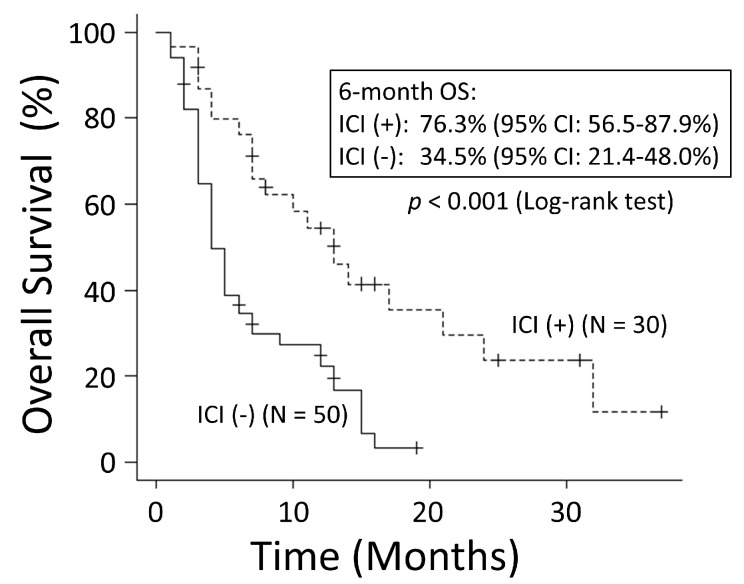
Patients with immune checkpoint inhibitors (ICIs) had significantly better overall survival (OS) than those without ICIs. CI: confidence interval.

There was no significant difference in OS rates between patients with NSCLC and those with SCLC (*p* = 0.249) (Figure 1). The good performance status (PS) group (PS ≤ 1) had a significantly better OS rate than the poor PS group (PS ≥2) (*p* < 0.001) (Figure 2). The 6-month OS rate of the good PS group was 59.4%, and its MST was 10 months. The 6-month OS rate of the poor PS group was 28.9%, and its MST was 4 months. When the patients were divided into the two groups based on their BED values, either greater than the median BED of 39 Gy or ≤39 Gy, there was no significant difference in the OS rates between the two groups (*p* = 0.693) (Figure 3). There was no significant difference in the OS rates according to the different irradiation sites (*p* = 0.122) (Figure 4). Additionally, there was no significant difference in the OS rates between groups with and without metastases at sites other than the irradiated sites (*p* = 0.104) (Figure 5). Patients treated with ICIs had significantly better OS rates than those not treated with ICIs (*p* < 0.001) (Figure 6). The 6-month OS rates for patients treated with and without ICIs were 76.3% and 34.5%, respectively, and the MSTs were 13 and 4 months, respectively.

**Figure 7:**
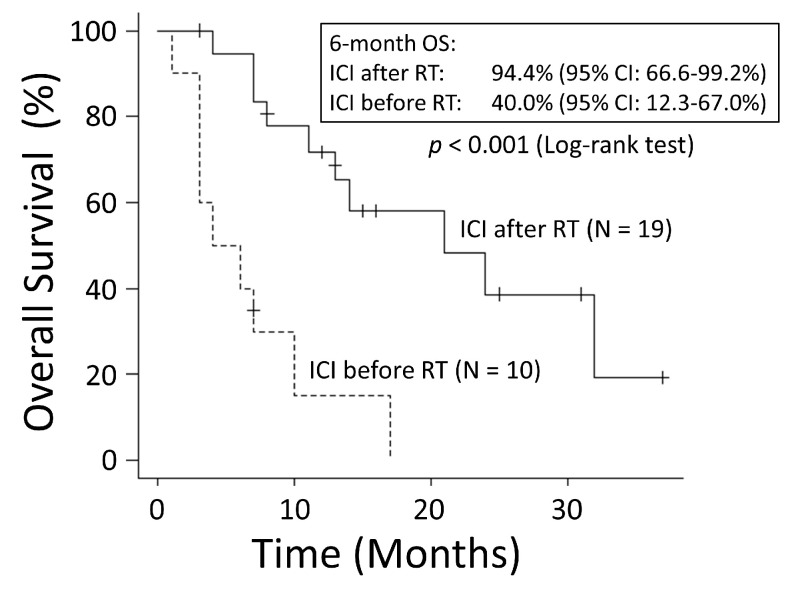
The group that used immune checkpoint inhibitors (ICIs) after radiation therapy (RT) had significantly better overall survival (OS) than the group that used ICIs before RT. CI: confidence interval.

**Figure 8:**
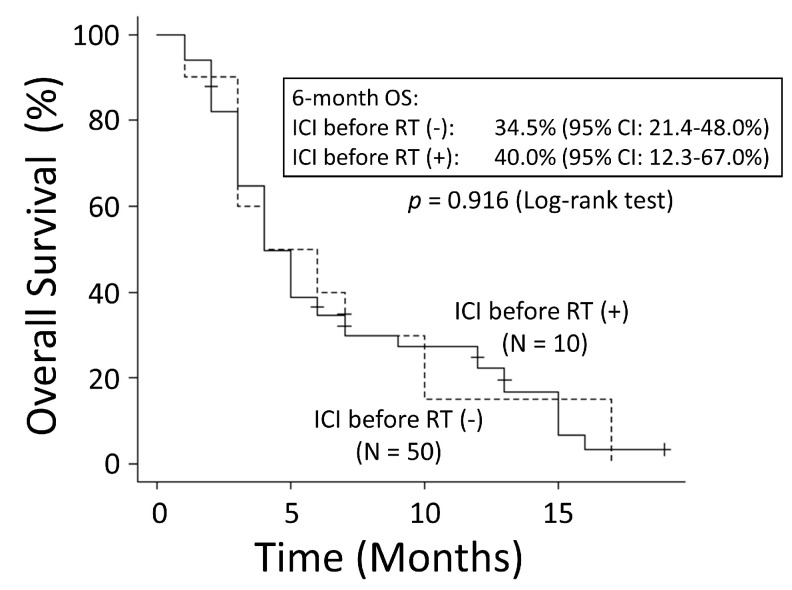
There was no significant difference in overall survival (OS) between the group that used immune checkpoint inhibitors before radiation therapy (RT) and the group that did not. CI: confidence interval.

To evaluate the effective timing of ICI use, only the group administered ICIs was compared with the group that received ICIs before RT and the group that received ICIs after RT. The patient characteristics within both groups are shown in Table 3. In the group that received ICIs before RT, the median time from the end of the ICI administration to the start of RT was 1 month (range: 0–13 months), and the median duration of the ICI therapy was 4 months (range: 1–13 months). In the group that received ICI therapy after RT, the median time from the end of RT to the start of ICI administration was 4 months (range: 0–25 months), and the median duration of the ICI therapy was 4 months (range: 1–20 months). The group that received ICIs after RT had a significantly better OS rate than the group that received ICIs before RT (6-month OS: 94.7% vs. 40.0%; MST: 21 months vs. 5 months, *p* < 0.001) (Figure 7). There was no significant difference in the OS rates between the group that used ICIs before RT and the group that did not (*p* = 0.916) (Figure 8).

The results of the univariate and multivariate analyses of the Cox proportional hazards model are shown in Table 4. In the univariate analysis, PS (0–1 vs. 2–4) and ICI use after RT were significant factors for OS (both *p* < 0.001). These two factors were also significant in the multivariate analysis (*p* = 0.032 and *p* < 0.001, respectively). 

## 4. Discussion

In experiments on tumor-transplanted mice, Deng et al. reported that RT plus anti-PD-L1 antibody treatment had a significantly higher antitumor effect than RT alone or anti-PD-L1 antibody treatment alone [15]. Building upon this research, a secondary analysis of the KEYNOTE-001 study for locally advanced or metastatic NSCLC showed significantly better progression-free survival and OS rates in patients with a history of RT prior to pembrolizumab than in those without a history of RT and who received pembrolizumab [16]. Additionally, in the PEMBRO-RT trial for metastatic NSCLC, patients who received pembrolizumab after stereotactic body RT tended to have significantly better response rates at 12 weeks than those who received pembrolizumab alone [17]. Moreover, although there was no significant difference, the OS was also better in patients who received pembrolizumab after RT than in those who received pembrolizumab alone. Furthermore, the usefulness of the combination of RT and ICIs has been reported many times [18,19,20,21,22,23]. While these reports indicate that ICIs have been used in combination with RT, or after RT, no studies have yet compared the efficacy of ICIs administered before vs. after RT. Interestingly, in our study, there was no difference in OS between patients who received ICIs before RT and those who did not. In contrast, patients who received ICIs after RT had significantly better OS than those who did not. This suggests that the use of ICIs after RT may improve OS, presumably due to the activation of antitumor immunity by RT. The direct DNA-damaging action of radiation and its indirect action via radicals are the conventionally known mechanisms of radiotherapeutic effects [24]. Recently, immunological mechanisms such as high-mobility group box 1 and calreticulin-mediated dendritic cell activation, cytotoxic T lymphocyte activation by enhancing major histocompatibility complex class I expression, and activation of the cyclic GMP-AMP synthase-stimulator of the interferon gene pathway have been elucidated [24,25,26,27,28]. At the same time, PD-L1 expression in tumors increases after RT, which has a suppressive effect on antitumor immunity. Thus, RT has both stimulating and suppressing effects on antitumor immunity. Therefore, the combined use of ICIs after RT is expected to mitigate the suppressive effect of antitumor immunity induced by RT.

Wang et al. reported that when RT and ICIs are combined, patients with a dose per fraction of 5 Gy or greater have significantly better OS than those with a dose per fraction of less than 5 Gy [29]. In addition, Welsh et al. reported that their group that received 50 Gy in 4 fractions (12.5 Gy per fraction) had a better response rate outside of the RT field than the group that received 45 Gy in 15 fractions (3 Gy per fraction) when pembrolizumab was used in combination with RT [30]. Moreover, it has been reported that the expression of INF-γ-associated genes after RT is associated with the response outside of the RT field [31]. Deng et al. also reported that IFN-γ-producing T cells increased in the lymph nodes of mice after IR + anti-PD-L1 treatment [15]. In our study, there was no difference in OS rates according to the dose per fraction. Notably, our study did not include cases with high doses per fraction, and the doses per fraction were relatively similar.

The limitations of this study are that it was a retrospective study, the number of cases was small, it included cases with various histological types, irradiation sites, and irradiation doses, and it did not consider the effects of cytotoxic chemotherapy. Systemic therapies such as cytotoxic anticancer agents and ICIs were used in many patients, and many patients used multiple regimens. The most commonly used regimens were cisplatin/carboplatin + etoposide in 21 patients, amrubicin in 15, S-1 in 14, pembrolizumab in 11, docetaxel in 10, carboplatin + paclitaxel in 9, cisplatin/carboplatin + pemetrexed + bevacizumab in 7, carboplatin + nanoparticle albumin-bound paclitaxel in 6, and cisplatin/carboplatin + pemetrexed + pembrolizumab in 6. Few female patients were enrolled in this study. We speculate that this was because the exclusion criteria included patients who used TKIs. East Asian women have a considerably high rate of EGFR mutation positivity [32,33,34], which may explain why many female patients were excluded because of TKI use.

Our results suggest that ICI administration after RT may prolong OS in patients with stage IV lung cancer. Future studies should investigate the optimal timing and duration of ICI therapy in this context.

## 5. Conclusions

In conclusion, ICI administration after RT may prolong OS in patients with stage IV lung cancer.

## Figures and Tables

**Table 1:** Patient characteristics (N = 80).

Age (Years)	Median (Range)	70 (43–88)
Sex	Male/female	69 (86.3%)/11 (13.8%)
ECOG performance status	0	15 (18.8%)
	1	40 (50.0%)
	2	13 (16.3%)
	3	9 (11.3%)
	4	3 (3.8%)
BED (α/β = 10) (Gy)	Median (range)	39.0 (27.3–72.0)
Irradiated site	Bone	44 (55.0%)
	Brain	21 (26.3%)
	Mediastinum	7 (8.8%)
	Cervical lymph node	4 (5.0%)
	Others	4 (5.0%)
Other metastases	Yes/no	64 (80.0%)/16 (20.0%)
ICI use	Yes/no	30 (37.5%)/50 (62.5%)
Follow-up period (months)	Median (range)	6 (1–37)

ECOG: Eastern Cooperative Oncology Group, BED: biologically effective dose, ICI: immune checkpoint inhibitor.

**Table 2:** Comparison of patient characteristics in patients who received ICIs and those who did not.

		ICI (+) (N = 30)	ICI (−) (N = 50)
Age (years)	Median (range)	70 (43–86)	71 (48–88)
Sex	Male/female	27 (90.0%)/3 (10.0%)	43 (86.0%)/7 (14.0%)
ECOG performance status	0	8 (26.7%)	7 (14.0%)
	1	16 (53.3%)	24 (48.0%)
	2	4 (13.3%)	9 (18.0%)
	3	1 (3.3%)	8 (16.0%)
	4	1 (3.3%)	2 (4.0%)
Histology	Adenocarcinoma	19 (63.3%)	12 (24.0%)
	Squamous carcinoma	7 (23.3%)	12 (24.0%)
	Small cell carcinoma	3 (10.0%)	18 (36.0%)
	Others	1 (3.3%)	8 (16.0%)
PD-L1 expression	≥25%	12 (40.0%)	2 (4.0%)
	<25%, 1%≥	6 (20.0%)	5 (10.0)
	<1% or unknown	12 (40.0%)	43 (86.0%)
BED (α/β = 10) (Gy)	Median (range)	39.0 (28.0–72.0)	39.0 (27.3–48.0)
Irradiated site	Bone	16 (53.3%)	28 (56.0%)
	Brain	6 (20.0%)	15 (30.0%)
	Mediastinum	3 (10.0%)	4 (8.0%)
	Cervical lymph node	3 (10.0%)	1 (2.0%)
	Others	2 (6.7%)	2 (4.0%)
Other metastases	Yes/no	24 (80.0%)/6 (20.0%)	40 (80.0%)/10 (20.0%)
Chemotherapy	Yes/no	30 (100%)/0 (0%)	35 (70.0%)/15 (30.0%)

ICI: immune checkpoint inhibitor, ECOG: Eastern Cooperative Oncology Group, PD-L1: programmed cell death-ligand 1, BED: biologically effective dose.

**Table 3:** Characteristics of patients who received ICIs (N = 29).

		ICIs after RT (N = 19)	ICIs before RT (N = 10)
Age (years)	Median (range)	71 (43–79)	66 (46–86)
Sex	Male/female	19 (100%)/0 (0%)	7 (70.0%)/3 (30.0%)
ECOG performance status	0	5 (26.3%)	2 (20.0%)
	1	12 (63.2%)	4 (40.0%)
	2	1 (5.3%)	3 (30.0%)
	3	0 (0%)	1 (10.0%)
	4	1 (5.3%)	0 (0%)
Smoking status	Current smoker	1 (5.3%)	3 (30.0%)
	Past smoker	4 (21.1%)	1 (10.0%)
	Never smoked	14 (73.7%)	6 (60.0%)
Histology	Adenocarcinoma	10 (52.6%)	8 (80.0%)
	Squamous carcinoma	6 (31.6%)	1 (10.0%)
	Small cell carcinoma	2 (10.5%)	1 (10.0%)
	Others	1 (5.3%)	0 (0%)
PD-L1 expression	≥25%	7 (36.8%)	5 (50.0%)
	<25%, 1% ≥	3 (15.8%)	2 (20.0)
	<1% or unknown	9 (47.4%)	3 (30.0%)
BED (α/β = 10) (Gy)	Median (range)	39.0 (28.0–72.0)	39.0 (28.0–43.8)
Irradiated site	Bone	13 (68.4%)	3 (30.0%)
	Brain	4 (21.1%)	2 (20.0%)
	Mediastinum	2 (10.5%)	1 (10.0%)
	Cervical lymph node	0 (0%)	2 (20.0%)
	Others	0 (0%)	2 (20.0%)
Period between RT and ICI therapy *	Median (range)	1 (0–13)	4 (0–25)
Duration of ICI therapy	Median (range)	4 (1–13)	4 (1–20)
Other metastases	Yes/no	13 (68.4%)/6 (31.6%)	10 (100%)/0 (0%)
Chemotherapy	Yes/no	19 (100%)/0 (0%)	10 (100%)/0 (0%)
No. of systemic therapy regimens	5	1 (5.3%)	0 (0%)
	4	1 (5.3%)	1 (10.0%)
	3	2 (10.5%)	5 (50.0%)
	2	12 (63.2%)	2 (20.0%)
	1	2 (10.5%)	2 (20.0%)
	0	1 (5.3%)	0 (0%)

ICI: immune checkpoint inhibitor, RT: radiation therapy, ECOG: Eastern Cooperative Oncology Group, PD-L1: programmed cell death-ligand 1, BED: biologically effective dose. * Time from the end of ICI administration to the start of RT or the end of RT to the start of ICI administration.

**Table 4:** Univariate and multivariate analyses for overall survival rates.

Variables		Univariate		Multivariate	
		HR (95% CI)	*p* Value	HR (95% CI)	*p* Value
Age (years)	<70 vs. ≥70	1.321 (0.7978–2.186)	0.280		
Pathology	NSCLC vs. SCLC	0.7261 (0.4084–1.291)	0.276		
BED (Gy)	≤39 vs. >39	1.133 (0.5896–2.179)	0.707		
Dose per fraction (Gy)	≤3 vs. >3	0.9796 (0.4809–1.996)	0.955		
Other metastases	Yes vs. no	1.704 (0.8595–3.378)	0.127		
ECOG performance status	0–1 vs. 2–4	2.587 (1.475–4.537)	<0.001	1.8560 (1.055–3.265)	0.032
ICI use after RT	Yes vs. no	0.2121 (0.09915–0.4538)	<0.001	0.2438 (0.112–0.531)	<0.001

HR: hazard ratio, CI: confidence interval, NSCLC: non-small-cell lung cancer, SCLC: small-cell lung cancer, BED: biologically effective dose, ECOG: Eastern Cooperative Oncology Group, ICI: immune checkpoint inhibitor, RT: radiation therapy.

## Data Availability

The datasets used and/or analyzed during the current study are available from the corresponding author on reasonable request.
